# ASSOCIATION OF LIVER STIFFNESS BY TRANSIENT ELASTOGRAPHY WITH MOBILITY DIFFICULTY IN OLDER ADULTS WITH NO CIRRHOSIS

**DOI:** 10.1093/geroni/igac059.1392

**Published:** 2022-12-20

**Authors:** Paulo H Chaves

**Affiliations:** Florida International University, Miami, Florida, United States

## Abstract

Growing experience with non-invasive ultrasound transient elastography (TE) offers now a major opportunity to improve understanding about the functional impact of non-advanced, asymptomatic liver dysfunction. We assessed the relationship of liver stiffness measurement (LSM) and controlled attenuation parameter (CAP) obtained by TE, surrogates for liver fibrosis and steatosis, respectively, with mobility difficulty, an early stage of the disablement process. Cross-sectional study utilizing National Health and Nutrition Examination Survey 2017-2018 data. Analytic sample (n=1,203) included participants aged 60 years and older without known cirrhosis with available LSM and CAP. Logistic regression assessed the odds of mobility difficulty (walking ¼ mile or climbing stairs) according to LSM and/or CAP, with adjustment for demographics, diseases, metabolic syndrome, body mass index, aminotransferases, gamma-glutamyl transferases, albumin, and platelets. LSM and CAP were linearly, positively associated with the probability of mobility difficulty. In the fully adjusted model, LSM, though not CAP, remained strongly associated with mobility difficulty. Those with LSM in the top (>7 kilopascals [kPa]) and intermediate quintiles (4-7 KPa) had higher odds of mobility difficulty; i.e., odds ratio (OR): 1.81; 95% confidence interval (CI): 1.15-2.86; p=0.01) and OR:1.45; 95%CI: 1.01-2.08; p=.046), respectively. LSM was independently associated with early mobility disability in older adults without known cirrhosis. A direct effect through sarcopenia promotion pathways might be speculated. Whether TE screening could be useful to identify adults with non-advanced liver fibrosis who might benefit from preventive interventions – e.g., diet and exercise for non-alcoholic fatty liver disease patients vis-à-vis mobility disability prevention remains to be tested.

